# Adenoid cystic carcinoma of Bartholin’s gland diagnosed after lung lobectomy: Review of the literature and a case presentation

**DOI:** 10.4274/tjod.galenos.2020.39225

**Published:** 2020-12-10

**Authors:** Seda Şahin Aker, Cevriye Cansız Ersöz, Fırat Ortaç

**Affiliations:** 1 Ankara University Faculty of Medicine, Department of Gynecologic Oncology, Ankara, Turkey; 2 Ankara University Faculty of Medicine, Department of Pathology, Ankara, Turkey

**Keywords:** Adenoid cystic carcinoma of the Bartholin’s gland, vulvar cancer, Bartholin gland carsinom

## Abstract

Bartholin’s gland was first identified in human female in 1675 by Caspar Bartholin. The Bartholin gland is composed of several epithelial types: The body is mucinous acini, the duct is predominantly transitional epithelium, and the orifice is the squamous epithelium. Primary carcinoma of the bartholin’s gland is an uncommon neoplasm. Adenoid cystic carcinoma (ACC) of bartholin gland carcinom is a rare variant of bartholin gland carcinoma, comprising 15% of all bartholin gland malignancies. ACC of the Bartholin’s gland is characterized by slow growth so recurrence and distant metastases can take a long period. So distant metastasis has been found in only a few cases to the lungs, liver, bone and brain. Here, we present the case of Bartholin’s gland ACC after four years follow up and presented with a lung metastasis.

## Introduction

The Bartholin gland was first identified in the human female body in 1675 by Caspar Bartholin^(1)^. The main function of the Bartholin gland is to secrete mucus to provide vaginal and vulvar lubrication. Each Bartholin gland is approximately 0.5 cm in size and drains mucous into a duct 2.5 cm long. The ducts open onto the vulvar vestibule at the four and eight o’clock positions on each side of the vaginal orifice. The Bartholin gland has different epithelial types: the body is mucinous acini, the duct is transitional epithelium, and the orifice is squamous epithelium^(2)^. Primary carcinoma of the Bartholin gland is an uncommon neoplasm. Bartholin gland carcinoma (BGC) comprises approximately 0.1 to 5% of all vulvar carcinomas and <1% of female genital malignancies^(3)^. BGC has many histologic types: adenocarcinoma, squamous, adenosquamous, transitional cell carcinoma, and adenoid cystic carcinoma (ACC)^(4)^. Adenocarcinomas and squamous cell carcinomas each account for approximately 40% and adenosquamous carcinomas account for approximately 5% of BGCs^(5)^. ACC of BGC is a rare variant, comprising 15% of all Bartholin gland malignancies and the first documentation of ACC was identified by Klobin in 1864^(6)^. In the literature, fewer than 90 patients have been described. ACC of the Bartholin gland is characterized by slow growth, so recurrence and distant metastases can take a long period. Only in rare cases are distant metastasis seen in the lungs, liver, bone, and brain. We present a case of ACC in a woman who presented with lung metastasis after four years of follow-up.

## Case Presentation

**Figure 1:**
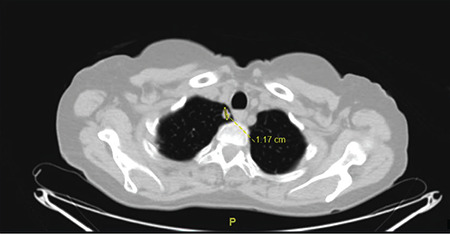
CT scan showed right upper lung lesion in diameter of 1,2*1,2 cm CT: Computed tomography

**Figure 2:**
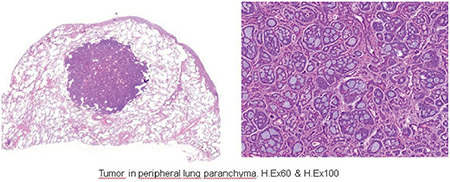
Tumor in peripheral lung paranchyma

**Figure 3:**
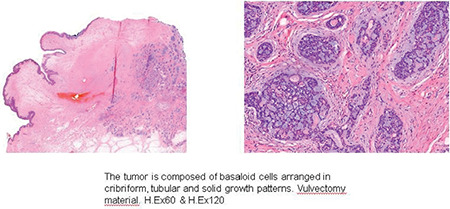
Tumor in vulvectomy material

In September 2014, a 58-year-old gravidity 5 parity 2 menopausal women presented with a palpable mass and vulval pain of the left Bartholin’s gland area. Under general anesthesia, the left Bartholin’s gland was excised with a Bartholin gland cyst prediagnosis. A pathologic examination revealed an ACC of the Bartholin gland, the tumor continued at the positive edge of surgery with negative perineural invasion. The unexpected malignant lesion was diagnosed and a scientific study was conducted for metastatic disease. Chest, abdomen, and pelvis computed tomography (CT) scans showed no metastatic disease. Then the patient underwent hemivulvectomy with left inguinofemoral lymph node dissection. After surgery, the pathology result showed a tumor on one side of the surgical margin and positive perineural invasion. The inguinofemoral lymph nodes were collected, all of which were tumor-free. The patient received no adjuvant treatment. We followed up the patient regularly and who showed no recurrence over a 4-year period. Forty-nine months after the surgery, she had chest pain and cough symptoms. A thorax CT scan was performed, which showed a right upper lung lesion in diameter of 1.2*1.1 cm (Figure 1). In September 2018, the patient underwent a left pulmonary wedge resection. The diagnosis was clarified with pathologic results. It showed a lung metastasis of ACC with no tumor in lymph nodes and margins are negative (Figure 2). A pathology examination showed columns of cells arranged concentrically around gland-like spaces filled with eosinophilic periodic acid-Schiff-positive material. In addition, the examination showed the characteristic tumor proliferation in a cribriform pattern composed of nests, hence, the presence of ACC metastasis. Two years earlier, when the first ACC was diagnosed, it showed similar speciality and was clarified with immunohistochemical characteristics. Tumour cells were widely positive with SMA, CD117, and p63, and focally positive with CK7 and calponin (Figure 3). The patient is now disease-free with 56 months of follow-up and stable disease.

## Discussion

In 1864, Klob was the first to describe BGC. It has various types such as adenocarcinoma, squamous, adenosquamous, transitional cell carcinoma, and ACC. Ten to fifteen percent of patients have ACC, which is histologically similar to adenocarcinoma of the salivary glands^(7)^. ACC of the Bartholin gland is extremely rare. Only 80 cases have been reported in the literature^(8)^. Common symptoms are a painless mass, pruritus, dyspareunia, burning sensation, vulvar pain, and abnormal bleeding. Initial misdiagnosis or delayed diagnosis may occur in up to 50% of patients, with incorrect diagnoses of Bartholin cysts or abscesses^(7)^. The most frequent symptoms is a progressive enlargement or swelling in the vulva; the patients often experience pain, which is probably due to tumor involvement of nerves^(9)^. In our case, the patient was misdiagnosed as having a Bartholin cyst and underwent Bartholin gland excision. The patient underwent reexcision and inguinofemoral lymphadenectomy because of incomplete surgery and margins with the tumor. The Bartholin gland was excised for the primary treatment. In women aged over 40 years diagnosed with Bartholin cysts, fine needle aspiration cytology is to exclude malignancy recommended^(10)^. The incidence of Bartholin gland tumors is highest among women in their 60s. The incidence of BGC in one series was 0.023 per 100,000 woman-years in premenopausal women and 0.114 per 100,000 woman-years in postmenopausal women^(11)^; however, ACC of the Bartholin gland shows a different age predisposition. ACC can be shown from the late 20s^(8)^. ACC of the Bartholin’s gland shares classic histologic features with ACC of the salivary gland. The tumor is composed of uniform, small cells arranged in cords and nests with a cribriform pattern, variable-sized cysts filled with an amphophilic or eosinophilic acellular basement membrane-like material. The tumor must be located in an appropriate anatomic location. Most tumors have perineural invasion, which is thought to contribute to its high local recurrence rate^(12)^. ACC of the Bartholin gland is characterized by slow growth, local recurrence, and distant metastases by intravascular spread may occur over a long period^(13)^. Distant metastasis of ACC is not common, it is very rare in the lung, brain, liver, and bone. Alsan et al.^(8)^ showed the most prevalent distant metastasis of ACC site was lungs, followed by liver, and rarely bone. Our patient had a distant metastasis after 4 years of follow-up.

Optimal surgical treatment for ACC of the Bartholin gland is not identified with guidelines. Radical vulvectomy ± inguinal lymphadenectomy and simple excision can both be performed. The most important point is to achieve a tumor-free margin. Yang et al.^(14)^ showed 68.9% of patients who had a simple excision had recurrence rate. Patients who underwent radical vulvectomy had a 42.9% recurrence rate; resection margins in the radical vulvectomy group were positive in 30% of the patients. The positive margin rate was 48% in the simple excision group. Hsu et al.^(15)^ presented two ACCs of Bartholin gland origin with lung metastasis; the first patient had a positive margin in pathologic examination and received postoperative adjuvant external beam radiotherapy, and distant metastasis (lung and bone) was found 42 months after the radiotherapy. The other patient who had tumor-free margins, had no adjuvant treatment. Fifty-nine months after the surgery she had lung metastases. Yoon et al.^(16)^ presented 5 cases of ACC of the Bartholin gland. Two had lung metastases. In the first case, a 54-year-old woman had right-side radical local excision + ipsilateral inguinal lymph node dissection + adjuvan radiotherapy. Resection margins and perineural invasion were positive. She had lung metastasis twice 7 and 8 months later and underwent metastasectomy with subsequent chemotherapy (paclitaxel and carboplatin) for the first metastasis and metastasectomy only for the second metastasis. After 71 months’ follow-up, she had stable disease. The second case was a 60-year-old woman who had left-side radical local excision and no adjuvant therapy. Resection margins and perineural invasion were positive. Local pelvic recurrence and distal metastases (lung) occurred in the patient, the local metastasis was removed. Lung metastases occurred 71.18 and 189 months after surgery and complete excision of the pulmonary lesion was not possible because of the number and size of the lesions. The patient had local excision and no adjuvant therapy. Resection margins and perineural invasion were positive. Local pelvic recurrence and distal metastases (lung) occurred in the patient, the local metastasis was removed. Lung metastases were at 71,179 and 189 months after surgery and complete excision of pulmonary lesion was not possible because the number and the size of lesions did not rise up.

After 224 months’ follow-up she had progressive disease^(16)^. In our case, like Yoon et al.^(16)^ cases, the patient had positive margins in pathologic examination, received no adjuvant treatment; she had lung metastases 49 months after surgery, and after 56 months follow-up, she has stable disease. When the distant metastasis was evaluated, it seemed to be related to margin positivity and perineural invasion. ACC of Bartholin’s gland is a slow-growing tumor, long-term survival is excellent according to Copeland et al.^(17)^ The 5-year progress-free interval is 47% and the 5-year survival rate is 71%. They are 38% and 50%, respectively, at 10 years, and 13% and 51% at 15 years. According to these results, it has been suggested to use 10- to 15-year-survival rates rather than 5-year survival rates for ACC of the Bartholin’s gland. The patient of BG-ACC metastasis of Inguinal lymph node take place seldomly. However, if it occurs, it is ipsilateral to the primary tumor. In our case, there was no lymph node metastasis. In the literature, the effect of lymphadenectomy on survival and prognosis is controversial. There is little information on the treatment of metastasis and the management depends on the location. In literature, there are no data to support chemotherapy to prevent distant metastasis. If a distant metastasis exists, chemotherapy treatment alternatives such as chlorambucil-adriamycin, methotrexate-dactinomycin, cyclophosphamide-adriamycin-cisplatin or cyclophosphamide can be used^(14)^. Various treatment modalities, including radiotherapy, may produce tumor regression. Publications have emphasized poor outcomes in patients with positive tumor margins even when treated with radiotherapy^(18)^. A prospective randomized study can provide the most powerful evidence for deciding the optimal treatment. Due to the rarity of ACC of the Bartolin gland, we can obtain data from reviews and large series.

ACC of Bartholin’s gland is a rare, vulvar malignancy with unpredictable biologic behavior. Physicians have to suspect it in women aged over 40 years with persistent Bartholin’s gland masses. There is no consensus on the treatment and the treatment modality must be tailored according to each patient. Radical local excision or radical vulvectomy ± lymphadenectomy seems to be the most suitable treatment. Positive surgical margins are a very important factor related to recurrence. Surgery, radiotherapy or chemotherapy regimens can be used for recurrence. Follow-up of ACC of the Bartholin gland has to be for a long period due its slow growth behavior.
